# Cognitive remediation for bipolar patients with objective cognitive impairment: a naturalistic study

**DOI:** 10.1186/s40345-017-0079-3

**Published:** 2017-04-13

**Authors:** J. Veeh, J. Kopf, S. Kittel-Schneider, J. Deckert, A. Reif

**Affiliations:** 1 Department of Psychiatry, Psychosomatic Medicine and Psychotherapy, Goethe-University, Heinrich-Hoffmann-Straße 10, 60528 Frankfurt, Germany; 2 Department of Psychiatry, Psychosomatics and Psychotherapy, University of Wuerzburg, Fuechsleinstrasse 15, 97080 Würzburg, Germany

**Keywords:** Bipolar disorder, Cognitive deficits, Cognitive remediation

## Abstract

**Background:**

Many bipolar patients (BP) are affected by cognitive impairments and reduced psychosocial function even after complete remission. In the present naturalistic study, we developed a tailored cognitive remediation program (CR) to evaluate the effect on objective and subjective neuropsychological performance, psychosocial functioning and quality of life.

**Methods:**

The CR program used a cognitive training software combined with group sessions to educate cognitive skills. 102 BP were screened by a neuropsychological test battery. Of those, 39 BP showed distinct cognitive impairments and 26 patients of them participated in the CR program for 12 weeks and then were retested. A matched control group consisting of 10 BP was measured at baseline and follow-up after three months (treatment as usual).

**Results:**

Within the training group, a significant improvement of cognitive performance after CR was observed in working memory (*p* = .043), problem solving (*p* = .031) and divided attention (trend, *p* = .065). The control group did not improve in any test measure. In addition, we detected a significant reduction of sub-depressive symptoms (*p* = .011) after the CR program. However, there was no change in psychosocial functioning and quality of life. Subjective cognitive complaints were not associated with objective test performance.

**Limitations:**

As we included exclusively BP with objectively assessed neurocognitive deficits, recruitment was difficult and subsequently we had a small sample size and were not able to implement a randomized group design.

**Conclusions:**

Our results suggest that BP with objective cognitive impairments could benefit from CR potentially with regard to executive functioning. Furthermore, there is preliminary evidence that CR could have a positive effect on subthreshold residual symptoms. However, to fully identify the possible implications of CR in bipolar disorder, larger randomized-controlled trials are needed in this new field of research.

**Electronic supplementary material:**

The online version of this article (doi:10.1186/s40345-017-0079-3) contains supplementary material, which is available to authorized users.

## Background

Impairments in processing speed, attention, working memory, verbal memory and problem solving are the most common cognitive deficits found in patients suffering from bipolar disorder (Mann-Wrobel et al. 2011; Martinez-Aran et al. 2000; Daglas et al. 2015). The severity and profile of dysfunctions varies; however, in 40–50% bipolar patients (BP), cognitive impairments are persistent and not simply related to an acute episode or affective symptoms (Aminoff et al. 2013; Iverson et al. 2011). Thus, even when a bipolar patient is stable regarding his emotional status, cognitive impairments can remain at least in a subgroup of patients (Volkert et al. 2014). Moreover, there is considerable evidence that persistent cognitive impairments are related to poor illness outcome and that some BP do not reach their former level of psychosocial functioning even during euthymia (Gilbert and Marwaha 2013; Bonnin et al. 2010). Therefore, interventions for bipolar patients should not only target affective symptoms, but also cognitive dysfunctions (Miklowitz 2011). However, to date no effective psychopharmacological options selectively targeting cognitive dysfunctions in psychiatric illnesses are available (Millan et al. 2012), and existing psychotherapeutic strategies for bipolar disorder focus primarily on emotions and maladaptive thoughts or behaviour (Meyer and Hautzinger 2013). A promising psychological treatment termed cognitive remediation (CR) specifically focuses on cognitive functioning and has turned to be an evidence-based treatment in patients with schizophrenia (Wykes et al. 2011). Given that BP have less severe, but similar cognitive deficits as compared to patients suffering from schizophrenia (Bora et al. 2010; Lewandowski et al. 2011), CR seems to be an option to improve cognitive disturbances that interfere with daily functioning in bipolar disorder as well (Anaya et al. 2012).

The overall aim of CR is the improvement of impaired cognitive functions in order to increase psychosocial functioning and quality of life (Vauth et al. 2000). The basic principle of CR is the concept of neuronal plasticity of the brain. It is based on two main approaches: restitution (stimulation of cognition by repetitive exercise) and compensation (e.g. memorization skills, use of environmental aids). There is evidence that cognitive training in healthy persons can increase, e.g. synaptogenesis and neurogenesis in the adult hippocampus and the efficiency of resting state metabolism in the frontal lobe (Valenzuela and Sachdev 2009). Therefore, CR could have positive effects on cognitive dysfunctions in patients with psychiatric disorders, who have difficulties in daily life due to cognitive deficits. Moreover, in the long-term, CR may even have a positive influence on the illness outcome of patients, as it might enable them to benefit from other interventions like psychoeducation and psychotherapy (Martinez-Aran et al. 2011). It has been demonstrated that noncompliance to psychopharmacological and psychological interventions is higher in patients with cognitive disturbances (Mago et al. 2014; Fagan et al. 2015), which might be a further indirect route how CR could improve outcome. Finally, there is evidence that an attentional training can foster neuronal networks that underlie executive control mechanisms, whereas emotion regulation could be improved indirectly by cognitive training (Raz and Buhle 2006).

However, to date, there is only little evidence of the efficacy of CR programs in patients with bipolar disorder. A small number of studies investigated the effects of CR (Demant et al. 2015; Naismith et al. 2010); while some provided mainly compensatory techniques (Deckersbach et al. 2010; Torrent et al. 2013), others only used a computerized and non-individualized cognitive training (Meusel et al. 2013; Preiss et al. 2013). Moreover, there were many differences in the applied software, the frequency and duration of training and the choice of outcome measures, which reduce comparability between previous studies. However, results of the first trials about CR were at least partially promising, but of limited informative value due to mixed groups of bipolar and unipolar depressive patients or the absence of a control group. An interesting randomized controlled trial (RCT) by (Torrent et al. 2013) conducted functional remediation (FR) in a large sample of BP. FR is a program which combines training in metacognition and psychosocial skills. The authors compared FR, psychoeducation and treatment as usual (TAU) and found an improvement of psychosocial functioning in the FR group, compared to TAU. However, there was no improvement of objective neuropsychological test performance; also, psychoeducation had a similar positive effect like FR. A recently published RCT (Demant et al. 2015) compared 12-week group-based CR to TAU and found neither an effect on neurocognitive measures nor on measures of psychosocial functioning and quality of life. To summarize, previous studies found initial evidence for the effectiveness of the applied CR programs in bipolar disorder; however, conclusions were preliminary, due to manifold methodological challenges.

The aim of the present quasi-experimental study thus was to examine the potential effects of a group-based CR program in a sample of euthymic bipolar outpatients who suffered of persistent subjective *and* objective cognitive deficits, measured beforehand in a standardized neurocognitive battery to stratify patients for further therapy. Previous studies included BP in the training irrespective of an actual reduction in neuropsychological or psychosocial functioning. In contrast, we conducted the CR program exclusively with BP showing distinct cognitive deficits in an extended test battery. We hypothesized that training participants show an improvement in neuropsychological test performance compared to the control group which consisted of BP who received TAU. Furthermore, we supposed that patients would improve in psychosocial functioning and quality of life after the CR program. As an additional outcome variable, we were interested in the validity of subjective complaints about cognitive impairments of BP and supposed that the CR training group reports a lower level of cognitive impairment after the training compared to the baseline.

## Methods

### Sample

Bipolar patients were recruited at the outpatient clinic of the Department of Psychiatry, Psychosomatics and Psychotherapy, University Hospital Wuerzburg. They were diagnosed by applying the structured interview for DSM-IV, axis I disorder (SCID) (First et al. 2002) by an experienced psychiatrist. All participants were euthymic, and it was ensured that patients were fully remitted for at least 3 months before testing (by evaluation of the patients itself and their treating psychiatrists in our outpatient clinic). Patients were 18–60 years old and native German speakers. Affective symptoms of BP were measured with clinical interviews and questionnaires. Criteria for euthymia were rating scores of MADRS <12 (Montgomery–Asberg depression ratings scale) (Montgomery and Asberg 1979) and YMRS <5 points (Young Mania rating scale) (Young et al. 1978). Furthermore, patients had to be on a stable medication for at least 4 weeks to qualify for testing. Exclusion criteria were previous head trauma, neurological illnesses, schizoaffective disorder, or substance abuse/dependency in the previous 2 years. Furthermore, BP were not able to participate if they had no internet connection and a computer at home, because this was required for the CR program. Patients who had received electroconvulsive therapy in the preceding six months were excluded as well.

### Measures

#### Clinical assessment

The following clinical characteristics (lifetime presence) of BP were recorded via structured interviews with the patients and a review of clinical records: number of episodes (depression, mania/hypomania, mixed episodes), bipolar subtype, age at illness onset, substance abuse (actual and life time), psychotic symptoms (life time), medication, and comorbid somatic or mental illnesses.

Current mood of all participants was recorded using the Positive and negative affect scale (PANAS) (Watson et al. 1988) prior to the neuropsychological testing. We applied the Montgomery–Asberg depression rating scale (MADRS) (Montgomery and Asberg 1979) and the Beck depression inventory, Second Edition (BDI-II) (Beck et al. 1996) to assess depressive symptoms. The Young Mania rating scale (YMRS) (Young et al. 1978) was used to assess (hypo-)manic symptoms.

Psychosocial functioning was assessed by the Mini-ICF-App Social Functioning Scale, a rating scale for limitations of activities and participation in psychological disorders (Linden and Baron 2005). The instrument is an observer-rated interview with thirteen domains of capacity in the past 2 weeks: adherence to regulations, planning and structuring of tasks, flexibility, competency, endurance, assertiveness, contact with others, group integration, intimate relationships, non-work activities, self-care, mobility, and competence to judge and decide. Each dimension is rated on a scale (1 = no impairment to 4 = total disability), total score can range from 0 to 52.

Quality of life was assessed with the brief version of the World Health Organization Quality of Life Questionnaire (WHOQOL-BREF; WHO 1998) (Angermeyer et al. 2000). This is a self-rating with a global Score of quality of life and four sub-scores for the following domains: physical health, psychological health, social relationships and environment.

The subjective perception of cognitive impairment was assessed with the German questionnaire FLEI (Beblo et al. 2010), which is a self-assessment test to measure subjective mental ability. The scales are ability in the areas of attention, memory and executive functions. These scores are combined to give an overall score for cognitive disturbances.

#### Neurocognitive assessment

Intellectual abilities were estimated via the German multiple-choice word test (MWT-B) (Lehrl et al. 1995). We conducted a 60-min neuropsychological testing, including a 10-min break. Five standardized tests were administered in a fixed order by the same trained, experienced clinical psychologist in a quiet room. Tests were selected based on evidence of sensitivity to cognitive deficits in bipolar disorder. The tests have good test–retest reliability and if possible parallel forms of the tests were used (CVLT, Tower of London). Except the verbal learning task, all tests were computerized. Individual reactions times (RTs) and number of errors or omissions were used as measures.

*Psychomotor speed and attention* was assessed by the compatible trials of the Stroop interference test where participants have to react to four colours as fast as possible with a press on the according coloured button (Puhr and Wagner 2011). Attention was measured using the subtest divided attention of the test battery of attentional performance (TAP) (Zimmermann 2011), where patients are asked to pay attention on visual and acoustic stimuli simultaneously. According to the test authors, the test–retest reliability of the measure is *r* = .64.

*Verbal memory* The California verbal learning test (CVLT) (Niemann et al. 2011) was administered to assess verbal learning and memory. Participants learn a list of 16 words on five trials and afterwards immediate and delayed free recall after 30 min is tested. The test–retest reliability of the measure is declared with *r* = .64.

*Executive functioning* Working memory was assessed by a 2-back task with numbers as targets (Subtest *Working Memory* of the TAP test battery) (Zimmermann 2011). According to the test manual of the TAP, the test–retest reliability of this subtest is *r* = .60. Furthermore, we analysed the incompatible trials of the *Stroop interference test* (Puhr and Wagner 2011) which is considered to measure selective attention and the ability to control automatic processes and response inhibition. Participants are asked to name the font colour of an incongruent printed colour word (incompatible trials). Therefore, depending on the condition, the test requires the participant to override or inhibit a highly automatic reading response. The test–retest reliability varies between *r* = .85 und *r* = .99. *Planning and problem solving* was assessed by the Tower of London Test (TOL) (Kaller et al. 2011), where participants have to move three beads (red, green, blue) on three wooden sticks of different length, mounted on a block base. For each problem, the beads have to be moved from the starting configuration to a target position in the minimum number of moves possible. The Cronbach Alpha (reliability) for this test is α = .71.

### Cognitive remediation program

The CR program consisted of a cognitive skills group and a computer-assisted program for cognitive rehabilitation. The group sessions took place weekly over 12 weeks and were moderated by a cognitive behavioural psychotherapist with expertise in bipolar disorder and neuropsychology (JV). Groups consisted of 4–6 patients, and the sessions lasted 90 min. The rationale for choosing a group setting and a treatment of 12 weekly sessions was that there are no indications that a longer duration will result in greater cognitive improvement and previous studies used similar concepts (Demant et al. 2015; Meusel et al. 2013; Preiss et al. 2013). In each session, patients were coached in several cognitive skills by practical exercises and worksheets (see Table 1). Within this part, the strategies were illustrated by examples of the daily life of the participants. After a short break of 10 min, patients took seat in a room with several computers, where they were asked to logging into the training software. Within this last part of the session, every patient practiced exercises of the rehabilitation program and was supported in the solution of problems by other group participants. Furthermore, the therapist helped patients to acquire strategies for difficult exercises and motivated patients by positive feedback.

**Table 1:** Summary of the cognitive remediation modules

Module	Target domain	Description of contents
I	Introduction in cognitive skills and learning style (1 session)	Explanation of the aims of the CR program and collection what are the expectations of the patients, to increase the intrinsic motivation and engagement of the patients
		Introduction and demonstration of the software
		Psychoeducation about cognitive functions (what cognitive skill is needed for a certain task) and identification of individual learning strengths/weaknesses and learning style (e.g. time factor, sensory style, organization of learning material, social component) of the patients
II	Attention (2 sessions)	Discussion of the differences between attention and concentration
		Patients are familiarized with the concept of mindfulness as possibility to build sustained attention, and a first exercise is performed
		Working out possibilities to reduce the level of distraction in daily life
III	Memory (2 sessions)	A theoretical model of learning and memory is explained
		Teaching of mnemonic techniques and simple encoding and compensatory strategies
		Assisting in the use of environmental aids (e.g. notes, checklists, electronic support)
IV	Problem solving and planning (4 sessions)	Strategies for being organized, time management and planning in daily life are introduced
		Discussion, how information can be gathered, organized and contrasted
		Strategy for problem solving (concept of D’Zurilla and Goldfried)
		Patients work at several exercises (e.g. complex logical problems) and are asked to verbalize the steps for successful solution (e.g. how to break down a task down into manageable parts)
V	Communication (2 sessions)	Discussion of how the social-emotional context affects cognitive functioning
		Promoting optimal conditions to be concentrated in the interaction with others
		Skills for social interaction, practical exercises (role play)
		Strategies to handle trouble finding words.
VI	Healthy living (1 session)	Talking about the importance of good sleep, healthy living (e.g. nutrition, alcohol) and physical exercise for mental abilities
		Summary of the learned strategy and transfer of the acquired cognitive skills to everyday life
		Encouraging patients to train their brain in future by challenges of daily life (e.g. reading newspaper regularly, to go without a shopping list)

We used the online-based software “HAPPYneuron Pro”, which is a platform for professionals for the effective delivery and management of cognitive remediation and rehabilitation programs in a patient-centric manner (http://www.scientificbraintrainingpro.com). It facilitates a specialized treatment for neuropsychiatric illness (Bowie et al. 2012; Bowie et al. 2013; Franck et al. 2013). In this program, exercises consist of interactive games, which are specially designed for targeted stimulation of key cognitive functions. The patients progress through a pre-defined set of exercise levels according to specific rules. By real-time feedback and highly positive reinforcement, participants are kept motivated. The training was personalized by a feature of the software that adapts the difficulty level of the training tasks, and provides detailed graphic and verbal performance feedback after each exercise. They had the possibility to log in into their individual account from home and were instructed to practice two times a week for 30 min. The therapist has detailed views about the patient’s compliance and progression through the program. Furthermore, the possibility of a home-based cognitive training gives a greater feasibility and cost-effectiveness.

### Procedures

BP were assessed by an extended neuropsychological test battery and received feedback about their test performance in detail. Patients who showed a performance below the average of the normative data in at least two (out of seven) test measures were informed about the CR training program and were asked if they would like to attend. If patients were not able to participate in the training, due to time or commuting constraints, they were asked to be part of the control group (no randomization) and the testing was repeated after 3 months without cognitive training.

The clinical and neuropsychological (pre-) measurement was administered 2–5 weeks before the CR training started. The post-measurement was organized within 2 weeks after the last CR group session training (respectively after 3 months in the control group). The therapy was conducted by JV; the observer-based measures were administered by a clinical psychologist, who was blinded to treatment allocation (JK). All patients received full medical care as appropriate during and after the intervention. The control group (TAU) received clinical care in our outpatient clinic, too, with one medical appointment per month.

### Statistical analysis

All statistical tests were performed using the Statistical Package for Social Sciences (SPSS), version 22.0 (SPSS Inc., Chicago, Illinois, USA). For all tests, the significance level was set at *p* < .05. Chi square tests and *t*-tests were used to compare demographic variables, medication and current mood by group. To compare the neuropsychological test performance, the mood ratings and the questionnaires (Mini-ICF App, WHOQOL-BREF and FLEI) we conducted a 2 × 2 analyses of variance (ANOVA) with between subject factor group (training participants vs. controls) and the within subject factor time (pre-post). Given that the data of the neuropsychological tests were not normally distributed, nonparametric post hoc tests were selected (Wilcoxon-*U* Test und Mann–Whitney *U* Test). To estimate the clinical effect of the results, effect sizes were calculated (Cohen’s *d*).

## Results

### Sample characteristics

**Fig. 1:**
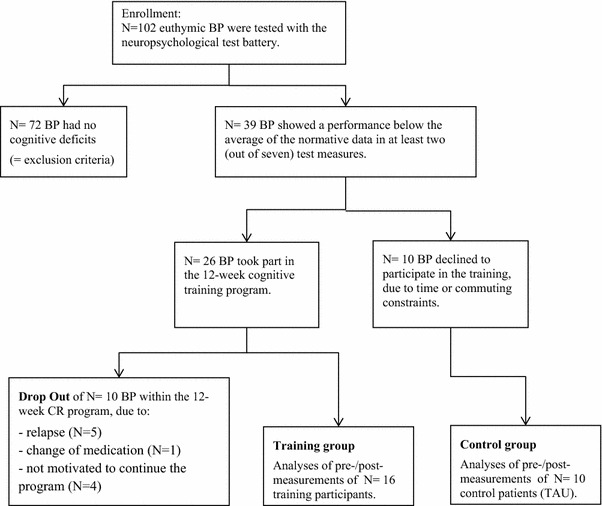
Flowchart of the recruiting process and drop-outs

Figure 1 and Table 2 display participant flow and characteristics, respectively. Within the sample of *N* = 102, we compared if BP with cognitive deficits (*N* = 39) differ from patients without deficits (*N* = 63) regarding age, sex, years of education, diagnosis subtype (I or II) and mood (MADRS, YMRS, PANAS) and did not find any differences (details can be found in the Additional file 1: Table S1). The final analyses of pre-/post-measurements could be conducted in 16 training participants in comparison to 10 TAU control patients. An intent-to-treat analysis could not be conducted as patients who dropped out could not be tested again after 3 months.

**Table 2:** Sociodemographic and clinical variables in the bipolar training group and the bipolar control group

	BP (training) (*N* = 16) N/M (SD)	BP (controls) (*N* = 10) N/M (SD)	*t*/χ^2^	*p* value (uncorr.)
Age	42.3 (12.2)	36.3 (12.3)	1.22	.235
Sex (f/m)	8/8	5/5	.00	.656
Years of education	11.8 (1.7)	11.6 (1.7)	.22	.829
Verbal IQ^a^	111.5 (9.6)	113.3 (11.5)	.43	.671
Bipolar Type I	9 (56.3%)	5 (50%)	.09	.536
Current mood				
PANAS^b^ PA^c^ (Pre)	29.5 (5.5)	29.6 (4)	−.05	.961
PANAS PA (Post)	30.7 (4.2)	29 (4.7)	.96	.349
PANAS NA^d^ (Pre)	12.6 (2.9)	12.1 (2.7)	.46	.652
PANAS NA (Post)	12 (2.1)	13.2 (2.3)	−1.37	.184
Medication				
Lithium	8 (50%)	9 (90%)	4.35	.045*
Other MS	4 (25%)	2 (20%)	.09	.580
Antipsychotics	8 (50%)	7 (70%)	1.01	.277
Antidepressants	10 (62.5%)	6 (60.5%)	.02	.609

*BP* bipolar patients, *MS* mood stabilizer (Lamotrigin, Carbamazepin, Valproat)

*p < 0.05

^a^Verbal IQ (German multiple-choice word test, MWT-B)

^b^Positive and negative affect scale

^c^Score positive affect

^d^Score negative affect

Demographic and clinical characteristics of the sample are presented in Table 2. There were no group differences regarding age, premorbid IQ and gender between groups. Participants’ actual mood (measured by PANAS score) showed no significant differences between groups and time point (pre vs. post). According to comorbid diseases, one patient of the training group suffered from comorbid ADHD and two (one each in the training and the control group) had a comorbid anxiety disorder. All patients were medicated; except for lithium, there were no differences between groups. None of the patients were treated with benzodiazepines or barbiturates.

### Changes in mood, psychosocial functioning and quality of life

The results of the 2 × 2 analyses of variance are presented in Table 3 and revealed a marginal main effect for time in sub-depressive symptoms (MADRS). Post hoc tests showed less depressive symptoms (MADRS) in the training group (*U* = −2.56, *p* = .011; *d* = 1.24) compared to the control group (*U* = −.29, *p* = .766). Other measures of mood (BDI-II and YMRS), psychosocial functioning (Mini-ICF-App) and quality of life (WHOQL-Bref) did not change. We did not find an association between mood, psychosocial functioning, quality of life and the neurocognitive test measures.

**Table 3:** Comparison of changes in mood, psychosocial functioning, life quality and subjective cognitive deficits (pre- vs. post-measurement) between training participants and control group

	BP (training) *N* = 16		BP (controls) *N* = 10		2 × 2 ANOVA					
					Time (d*f* = 1,22)		Group (d*f* = 1,22)		Time*group (d*f* = 1,22)	
	Pre M (SD)	Post M (SD)	Pre M (SD)	Post M (SD)	*F*	*p*	*F*	*p*	*F*	*p*
Mood ratings										
MADRS	7.9 (2.7)	4.9 (2.3)	6.1 (2.9)	6 (2.9)	4.09	.*054*	.25	.622	3.58	*.071*
BDI-II	8.6 (5.2)	7 (4.5)	5.6 (4.9)	4.6 (3.9)	1.83	.190	2.4	.135	.05	.822
YMRS	.75 (.9)	.75 (.8)	.7 (1.6)	.8 (1)	.02	.879	.00	1.00	.02	.879
Functioning										
Mini-ICF-App (global score)	10.1 (3.8)	8.6 (3.5)	8.1 (3.3)	7.9 (3.8)	2.03	.168	1.01	.326	1.16	.293
WHOQL-Bref (global score)	61.6 (17.9)	62.5 (18.8)	68.1 (9.1)	62.3 (24.8)	.06	.802	.45	.508	.22	.642
Subjective cognitive deficits										
FLEI (global score)	55.8 (25.9)	55.7 (25.3)	39.7 (27)	54.1 (26.9)	1.81	.192	.79	.382	1.51	.233
FLEI (attention)	20.1 (8.8)	19.8 (8.9)	15.8 (10.4)	16.1 (8.8)	.00	.993	1.19	.288	.03	.873
FLEI (memory)	18.8 (8.9)	18.7 (7.3)	18.3 (7.3)	19.9 (9.9)	.28	.607	.10	.922	.32	.576
FLEI (executive functions)	15.9 (8.0)	15.5 (8.6)	13.8 (8.9)	14.3 (8.3)	.00	.980	.28	.599	.09	.773

*BP* bipolar patients, *MADRS* Montgomery–Asberg depression rating scale, *BDI-II* Beck depression scale, *YMRS* Young Mania rating scale, *GAF* global assessment of functioning, *Mini-ICF-App* rating for limitations of activities and participation in psychological disorders (social functioning scale), *WHOQL-Bref* World Health Organization Quality of Life questionnaire, *FLEI* questionnaire to measure subjective mental ability, *df* degrees of freedom

### Subjective complaints about cognitive impairments and association with objective neuropsychological deficits

Patients were asked about their subjective perception of cognitive disturbances by the German questionnaire FLEI. We compared the sub-scores of our sample with the mean scores of normative controls (see evaluation study; Beblo 2010). The training group as well as the control group showed significantly higher global sum-scores in the FLEI compared to normative controls (*N* = 97; *M* = 29.1, SD = 18.7). Therefore, we confirmed our hypotheses that our sample of BP complained about impaired mental abilities. However, these subjective statements (FLEI global sum-score and scores of subscales) did not correlate with objective neurocognitive test measures at the pre- and post-measurements, except the number of omission in the working memory task (*r* = −.386, *p* = .057) at baseline. Moreover, the self-rating of cognitive complaints did not change within three months, neither in the training group, nor in the bipolar control group (see Table 3).

### Changes of the neurocognitive performance of training participants compared to controls

Neuropsychological test performance of the two samples (pre- vs. post-measurements) is presented in Table 4. We found a significant main effect for time (pre vs. post) in the test assessing *delayed recall (CVLT)*. Furthermore, there was a trend in *immediate recall (CVLT), divided attention* and *working memory*. A significant main effect for group (training participants vs. controls) was revealed in *working memory* and *problem solving* (Tower of London).

**Table 4:** Changes of the neuropsychological test performance (pre–post) of the bipolar training group compared to the bipolar control group

	BP (training) *N* = 16		BP (controls) *N* = 10		2 × 2 ANOVA						Post Hoc Wilcoxon-*U* test
					Time (*d*f = 1, 22)		Group (*d*f = 1, 22)		Time*grou*p* (*d*f = 1, 22)		
Cognitive domain	Pre M (SD)	PostM (SD)	Pre M (SD)	Post M (SD)	*F*	*p*	*F*	*p*	*F*	*p*	(Change pre vs. post)
Psychomotor speed											
Stroop compatible words (RT)	833 (113)	797 (124)	803 (112)	780 (91)	2.77	.110	.28	.602	.13	.718	BP_T_–, BP_C_–
Stroop compatible Colours (RT)	766 (97)	753 (123)	766 (101)	767 (102)	.02	.883	.01	.921	.24	.629	BP_T_–, BP_C_–
Attention											
Divided attention (omissions)	3.6 (5)	2.1 (2.4)	3.3 (2.5)	2.7 (2.3)	3.52	*.073*	.02	.904	.59	.448	BP_T_↑, BP_C_–
Memory											
CVLT (total verbal learning)	51.9 (8.6)	58 (10.7)	55.6 (6.5)	62.2 (9.1)	15.39	.001**	1.19	.286	.03	.863	BP_T_–, BP_C_–
CVLT (immediate recall)	11.7 (1.8)	12.4 (2.3)	11.8 (2.6)	13.1 (2)	3.96	*.059*	.25	.624	.32	.579	BP_T_–, BP_C_–
CVLT (delayed recall)	11.7 (2.1)	13.4 (2.1)	11.4 (3.1)	13.3 (2.1)	10.7	.003**	.09	.760	.01	.911	BP_T_↑, BP_C_–
Executive functions											
Working memory (omissions)	3.1 (2.1)	1.7 (1.7)	4.4 (1.8)	4.2 (1.7)	3.38	*.079*	9.73	.005**	1.95	.176	BP_T_↑, BP_C_–
Cognitive flexibility (errors)	2.1 (2.6)	.9 (1.1)	1.8 (1.7)	1.9 (1.2)	1.50	.233	.43	.521	2.09	.161	BP_T_–, BP_C_–
Stroop incompatible (reading)	109 (88)	132 (92)	199 (111)	165 (78)	.11	.747	3.13	.090	2.83	.106	BP_T_–, BP_C_–
Stroop incompatible (naming)	188 (207)	248 (234)	122 (94)	111 (124)	1.18	.290	1.78	.195	2.47	.130	BP_T_–, BP_C_–
Tower of London (problem solving)	6.4 (1.9)	8.4 (3.1)	5.9 (2)	5.4 (2.7)	1.38	.252	5.10	.034*	3.32	*.082*	BP_T_↑, BP_C_–

*BP* bipolar patients, *BP*_*T*_ training group, *BP*_*C*_ control group, ↑ improvement from pre- to post-measurement, – no change in performance from pre- to post-measurement, *CVLT* California verbal learning test, RT reaction time (ms)

*p < 0.05; **p < 0.01

In none of the test measures, the interaction (time*group) was significant. However, due to our a priori hypotheses and the fact that the neuropsychological data were not normally distributed, we conducted nonparametric post hoc tests. The results of the Wilcoxon-*U* tests showed a marginal improved performance of training participants in divided attention (*U* = −1.84, *p* = .065), whereas the bipolar controls did not improve (*U* = −.86, *p* = .389). The training group improved in *delayed recall (CVLT)* (*U* = −2.83, *p* = .005), but not in *immediate recall* (*U* = −1.54, *p* = .123). The number of memorized words in the CVLT did not improve in the control group: *immediate recall* (*U* = −1.05, *p* = .293) and *delayed recall* (*U* = −1.36, *p* = .176). The performance in the subtest working memory improved in the CR group (*U* = −2.03, *p* = .043), but not in controls (*U* = −.30, *p* = .762). Furthermore, training participants solved more problems in the test Tower of London after the training (*U* = −2.20, *p* = .031), while controls did not show a change in performance (*U* = −.57, *p* = .572). In addition, we conducted post hoc Mann–Whitney *U* tests to test if the groups differed from each other before and after the training. At baseline measurements (pre), the performance in the test battery of the training group did not differ from the performance of the control group, except the incompatible trial of the Stroop Test, Reading (*U* = 38.5, *p* = .031). At the post-measurement after CR, we found that the training group showed a significantly better performance in the subtest *working memory* (*U* = 22.0, *p* = .002) and *Tower of London* (*U* = 26.5, *p* = .008) compared to the control group (for more details about post hoc tests, see Additional file 2: Table S2 and Additional file 3: Table S3).

**Fig. 2:**
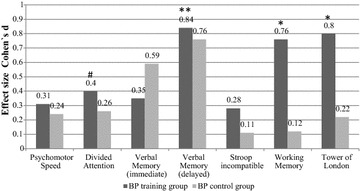
Effect sizes (Cohens’s *d*) of the change from pre- to post-measurement in the training group and the control group. (# Trend; *p < .05; **p < .01)

Figure 2 illustrates the effect sizes of the pre–post measurement and demonstrates medium to high effect sizes for the changes of the test performance of the CR group in the tests *divided attention, verbal memory, working memory* and *problem solving* In the verbal memory task, the control group improved their performance as well, although this was not significant. However, the fact that controls showed mainly marginal improvements indicates learning and practice effects, even though the test measures have excellent test–retest reliability scores (see “Neurocognitive assessment” section).

## Discussion

In the present naturalistic study, we investigated whether euthymic BP with objective cognitive deficits could benefit from a manualized cognitive remediation program. Hence, this is to our knowledge the first study about CR in bipolar disorder that conducted a CR training that was stratified according to cognitive deficits. Our results provide preliminary evidence of the efficacy of CR in bipolar disorder; we found an improved neurocognitive performance with medium to large effect sizes in the training group compared to the control group especially in executive functions. Furthermore, we found a significant improved verbal memory in the training group compared to the control group. However, the effect size of the improved verbal learning score is also very high in the control group and therefore clinical relevance of this result should be interpreted with caution. Our results are in line with previous CR trials in bipolar disorder reporting improvements in neurocognitive performance (Naismith et al. 2010; Meusel et al. 2013; Preiss et al. 2013). Therefore, it seems possible to improve cognitive functions by a specialized CR program.

However, there are conflicting studies which did not find an effect of the applied training on the neuropsychological test performance (Demant et al. 2015; Torrent et al. 2013). Though, it should be noted that except the study of Demant et al. (2015), previous studies did not define neuropsychological improvement as main outcome variable, instead functional outcome was measured (e.g. Deckersbach et al. 2010; Torrent et al. 2013). Nevertheless, one problem for nonsignificant effects of some previous studies could be the inclusion of BP irrespective of *objective* neuropsychological deficits, which is a highly important factor especially considering the hypothesis of a “cognitive deficits subgroup” of BP (Volkert et al. 2014). If patients do not display cognitive deficits at study entry, no further room for improvement can be assumed (Demant et al. 2015). There might be a ceiling effect depending on the premorbid level of performance, as patients who for example already perform in the normative range can hardly improve their test performance higher than the average of the normal population. To our knowledge, our study is the first that *exclusively* included a cognitive deficit subgroup of patients, showing impairments in at least two neuropsychological domains. Another limitation of previous studies was that CR was provided to acutely ill patients or during partial remission, where cognitive disturbances are a common symptom. As most cognitive deficits completely vanish in remission after an adequate antidepressant treatment, we suggest that CR should be provided in the context of an overall rehabilitation program specifically to patients who are stabilized for a longer period of time (Medalia et al. 2009). Instead, cognitive deficits during an acute episode should be treated by an optimized pharmacotherapy.

### Improvement of sub-depressive symptoms

We also found a positive effect of the CR program on the mood of the patients, in that the training participants had a significant reduction in sub-depressive symptoms (MADRS) compared to the control group. This is in line with several previous studies which demonstrated a reduction of depressive symptoms in participants of a CR training (Anaya et al. 2012; Deckersbach et al. 2010; Preiss et al. 2013; Sole et al. 2014). Furthermore, recent research revealed that BP frequently suffer from persistent residual mood symptoms, despite being stable for at least 6 months (Vieta et al. 2008). At first glance, it seems surprising that CR improves subthreshold depression, given that CR is not designed to target affective symptoms. However, a look at the content of the CR program (see Table 1) reveals many similarities with general psychotherapeutic strategies for depressive disorders. First of all, the group setting provides a sense of relatedness among group members and increases motivation and engagement. The weekly group sessions and the homework exercises offer a daily structure, social support and the possibility to exchange coping strategies. Secondly, several elements of cognitive behavioural therapy are part of the training, e.g. the use of self-instructions, positive self-enforcement, metacognitive techniques and strategies to support social interaction and problem solving (Hautzinger 2003). Therefore, it seems that CR helps to enhance self-efficacy and empowerment by breaking down the cyclical downward spiral of feeling incompetent. If patients succeed in the tasks of the CR program, they could be motivated to cope with the various tasks of their daily life, which is an important step in the recovery process. Interestingly, the significant reduction of sub-depressive symptoms solely becomes apparent in the observer-based rating MADRS. However, even in the self-rating questionnaire BDI-II, we found a marginal, though clinical, meaningful improvement of 1.6 points (Cohens *d* = .34). Given that sub-depressive symptoms during clinical remission have been described as an important factor that may contribute to impairment in psychosocial functioning and life quality (Gilbert and Marwaha 2013), CR could have the potential to improve overall functioning and well-being in patients in partial remission. Furthermore, previous study results indicate that remitted BP have cognitive impairments because they are not fully remitted (Volkert et al. 2014) and there is evidence that subsyndromal depression and cognitive deficits have reciprocal (negative and positive) influences on each other (Bonnin et al. 2012; Weinstock and Miller 2010). Hence, the improvement of sub-depression could be seen as a positive effect and CR should be applied especially for BP suffering from residual symptoms.

### Effect of CR on psychosocial functioning, life quality and subjective perception of cognitive deficits

However, contrary to some previous trials about CR in bipolar disorder (Deckersbach et al. 2010; Torrent et al. 2013), we could not confirm our hypotheses that psychosocial functioning and life quality improve by a cognitive rehabilitation program. We found only a marginal change in social and occupational functioning in the training group compared to the control group. Possibly, the changes in psychosocial functioning were not significant due to the small sample size, or the applied interviews and questionnaires (Mini-ICF-App and WHOQL-Bref) are not sensitive enough to reveal changes in daily life functioning and life quality directly after the completion of the CR training sessions. It is feasible that a transfer of an improved cognition and the application of the learned cognitive skills need more time to be measurable. Hence, future studies should conduct a long-term evaluation of CR after for example three months of the training.

As tertiary outcome measures, we were interested in patients’ subjective evaluation about cognitive disturbances. At the end of the group sessions, patients indicated that they had felt more mentally fresh and alert. However, in the structured questionnaire FLEI, we could not demonstrate that patients complain less cognitive impairments after the training. This is in contrast with the study of (Demant et al. 2015), who found an improvement in subjective sharpness and mental acuity in a self-assessment instrument. One reason for this negative result could be an attentional bias, in that patients became very sensitized to their individual cognitive disturbances due to the salience of the subject during the CR skills group. In the sessions, the therapist very often had to normalize subjective complaints of patients, e.g. in that it is normal that one sometimes has trouble to find a word under stress or to remember names of persons after meeting them only once. Interestingly, we found no significant relationship between subjectively reported cognitive complaints (FLEI) and objectively assessed neuropsychological test performance. This is in accordance with other reports of such a discrepancy (Burdick et al. 2005; Martinez-Aran et al. 2005), and may reflect a general problem of self-perception in patients with mood disorders in terms of a tendency for pessimism and self-deprecation (Reid and Maclullich 2006). On the other side, the neuropsychological test measures may lack the ecological validity necessary for tapping experiences of cognitive problems in everyday life (Beblo and Exner 2010; Lange and Tucha 2010). Moreover, the test situation per se is different from one’s daily life at work or at home. During a neuropsychological assessment, which is only temporary, one is usually able to muster the effort and motivation to perform the tests, while it is more difficult to concentrate when one is alone at home with several social and emotional distractors.

### Limitations

The results of the present naturalistic study should be interpreted with caution because due to the very small sample size which turned out to provide suboptimal statistical power for the outcome analysis. We had problems in recruitment of euthymic BP with distinct (objective) cognitive deficits, and it was not possible to conduct a controlled randomized trial within this quasi-experimental design. Furthermore, several patients dropped out during the training. The intent-to-treat sample consisted of 26 BP with cognitive deficits, although 102 euthymic BP were screened with a neuropsychological test battery. The drop-out/loss-to-follow-up rate was 38.5% (for reasons see Fig. 1). However, the literature about CR or psychotherapy in general in psychiatric disorders reported similar drop-out rates: Torrent et al. (2013) reported 28.6% and Preiss et al. (2013) even 48% drop-out rate. Another limitation of the present longitudinal study is the possibility of learning and practice effects which could bias the improved performance in the post-measurement. Given that the training group worked for 12 weeks with the training software, they possibly become more used to computer tests and reaction time tasks. To control this in future, it would be recommended to use an “active” control group, in that controls are asked to do for example online games at the computer. Moreover, we unfortunately were not able to assess long-term effects of the training, as we had only one post-measurement after three months. In addition, a limitation of the study is the combination of the training of cognition (restitution) and the training of compensatory skills. Due to this, it is not clear which part of the training had induced the improved performance of training participants. Therefore, we suggest for future studies to investigate restorative and compensatory approaches of CR separately and to compare it to the combination of training elements. Moreover, it would be very interesting to assess the satisfaction and engagement of the training participants in a structured way (questionnaires etc.) to improve the training for future trials. Finally, the analyses of the mood self-ratings demonstrated that, despite recruitment of remitted BP, participants had subsyndromal depressive symptoms. This might have influenced the findings, and therefore, it is not clear if full remission is necessary for cognitive benefits of an intervention per se.

### Outlook

Based on these points of criticism, future research is warranted to investigate how the efficacy of CR in bipolar disorder could be maximized. The identification of the target group and possible moderator and mediator variables would be helpful to apply CR more differentiated. For this purpose, it would be helpful to develop manualized CR programs and to evaluate them in different languages, to ensure comparability of different study results. To date, it is not clear if CR leads to restoration of cognitive functions or merely to a compensation of altered neurobiological functions. Hence, it would be interesting to examine the neurobiological changes in association of CR (Meusel et al. 2013). Furthermore, future studies could try to combine CR with neurostimulation techniques like transcranial direct current stimulation (tDCS), repetitive transcranial magnetic stimulation (rTMS) or neurofeedback, as there is evidence that such methods can stimulate cognition (Kuo et al. 2014; Tortella et al. 2014; Heinrich et al. 2007; Monastra 2005). Moreover, future trials could combine CR with psychopharmacological “cognitive enhancers” like d-cycloserine (Breitborde et al. 2014), cholinesterase inhibitors like Galantamine-ER (Iosifescu et al. 2009), or erythropoietin (EPO) (Miskowiak et al. 2016).

### Conclusions

The present naturalistic study indicates that CR could positively effect neurocognitive performance like attention, working memory and problem solving in bipolar disorder. Furthermore, our results give preliminary evidence for the potential of CR to reduce sub-depressive symptoms in patients who are partially remitted. Therefore, we suggest that CR should be provided exclusively to patients with objective cognitive deficits as the subjective evaluation of cognitive impairments is not associated with objective test performance. However, due to the limitations of our study design, further larger RCTs are needed using objective measures as inclusion criteria for applying CR in bipolar patients. To summarize, further work is needed to determine and to increase the potential effectiveness of CR in bipolar disorder, or in general in mental disorders.

## Additional files

**Additional file 1: Table S1.** Sociodemographic and clinical variables in the deficit subgroup*and non-deficit subgroup.

**Additional file 2: Table S2.** Comparison of the Pre- and Post-Measurement in the bipolar training group and the bipolar control group.

**Additional file 3: Table S3.** Comparison of the bipolar training group and the bipolar control group at the first and second measurement (pre and post testing).
