# Supplementary material for: Cognitive remediation for bipolar patients with objective cognitive impairment: a naturalistic study
Source: Int J Bipolar Disord. 2017 Apr 13;5:8. doi: 10.1186/s40345-017-0079-3 (PMC5389951; doi:10.1186/s40345-017-0079-3)
Supplement: Supplementary file 1 — Additional file 1: Table S1. Sociodemographic and clinical variables in the deficit subgroup*and non-deficit subgroup. [file 40345_2017_79_MOESM1_ESM.docx]

Table S1. Sociodemographic and clinical variables in the deficit subgroup*

and non-deficit subgroup

|  | **BP with deficits**  **(N= 39)**  N / M (SD) | **BP**  **no deficits (N= 63)**  N / M (SD) | ***t* / χ^2^** | ***p* value (uncorr.)** |
| --- | --- | --- | --- | --- |
| Age | 41.2 (10.9) | 38.6 (10.7) | 1.17 | .246 |
| Sex (f/m) | 23/16 | 36/27 | 0.03 | .856 |
| Years of education | 11.7 (1.6) | 11.8 (1.5) | -0.26 | .979 |
| Verbal IQ^1^ | 114.4 (13.8) | 113.7 (12.0) | 0.24 | .814 |
| Bipolar Typ I | 23 (59%) | 32 (51%) | 0.65 | .421 |
| *Mood* |  |  |  |  |
| MADRS | 6.7 (3.6) | 6.2 (2.9) | 0.89 | .376 |
| YMRS | 1.4 (2.4) | 1.1 (1.1) | 0.66 | .511 |
| PANAS PA | 29.9 (6.9) | 30.5 (5.2) | -0.45 | .652 |
| PANAS NA | 12.8 (3.6) | 12.1 (2.2) | 1.20 | .232 |

BP= Bipolar patients

* patients who performed below the average of the normative data in at least two (out of seven) test measures

^1^ verbal IQ (German multiple-choice word test, MWT-B)

^2^ Positive and Negative Affect Scale

^3^ Score Positive Affect

^4^ Score Negative Affect
