# Supplementary material for: Cognitive remediation for bipolar patients with objective cognitive impairment: a naturalistic study
Source: Int J Bipolar Disord. 2017 Apr 13;5:8. doi: 10.1186/s40345-017-0079-3 (PMC5389951; doi:10.1186/s40345-017-0079-3)
Supplement: Supplementary file 2 — Additional file 2: Table S2. Comparison of the Pre- and Post-Measurement in the bipolar training group and the bipolar control group. [file 40345_2017_79_MOESM2_ESM.docx]

Table S2. Comparison of the Pre- and Post-Measurement in the bipolar training group and the

bipolar control group

|  | **BP training group** | | **BP control group** | |
| --- | --- | --- | --- | --- |
| **Pre vs. Post-testing** | ***Wilcoxon-U test*** | ***p*** | ***Wilcoxon-U test*** | ***p*** |
| Stroop compatible Reading (RT) | -1.59 | .112 | -0.47 | .635 |
| Stroop compatible Naming (RT) | -1.14 | .256 | -0.29 | .767 |
| Divided Attention (omissions) | -1.84 | **.065** | -0.86 | .389 |
| CVLT (immediate recall) | -1.54 | .123 | -1.05 | .293 |
| CVLT (delayed recall) | -2.83 | **.005**** | -1.36 | .176 |
| Working Memory (omissions) | -2.03 | **.043*** | -0.30 | .762 |
| Stroop incompatible Reading (RT) | -1.53 | .125 | -1.54 | .123 |
| Stroop incompatible Naming (RT) | -1.90 | **.057** | -0.18 | .859 |
| Tower of London (Problems Solved) | -2.16 | **.031*** | -0.57 | .572 |

BP= bipolar patients, CVLT= California Verbal Learning Test, RT= reaction time
