# Supplementary material for: Cognitive remediation for bipolar patients with objective cognitive impairment: a naturalistic study
Source: Int J Bipolar Disord. 2017 Apr 13;5:8. doi: 10.1186/s40345-017-0079-3 (PMC5389951; doi:10.1186/s40345-017-0079-3)
Supplement: Supplementary file 3 — Additional file 3: Table S3. Comparison of the bipolar training group and the bipolar control group at the first and second measurement (pre and post testing). [file 40345_2017_79_MOESM3_ESM.docx]

Table S3. Comparison of the bipolar training group and the bipolar control group at the first

and second measurement (pre and post testing)

|  | **Pre-Testing** | | **Post-Testing** | |
| --- | --- | --- | --- | --- |
| **BP training- vs. control group** | ***Mann-Whitney-U test*** | ***p*** | ***Mann-***  ***Whitney-U test*** | ***p*** |
| Stroop compatible Reading (RT) | 65.5 | .452 | 70.0 | .934 |
| Stroop compatible Naming (RT) | 76.5 | .856 | 61.5 | .559 |
| Divided Attention (omissions) | 64.5 | .421 | 65.0 | .452 |
| CVLT (immediate recall) | 77.0 | .897 | 51.5 | .452 |
| CVLT (delayed recall) | 76.0 | .856 | 59.5 | .787 |
| Working Memory (omissions) | 56.0 | .220 | 22.0 | **.002**** |
| Stroop incompatible Reading (RT) | 39.5 | **.031*** | 57.5 | .419 |
| Stroop incompatible Naming (RT) | 67.5 | .517 | 43.5 | .108 |
| Tower of London (Problems Solved) | 59.0 | .286 | 26.5 | **.008**** |

BP= bipolar patients, CVLT= California Verbal Learning Test, RT= reaction time
